# A multilevel model of the help-seeking behaviors among adolescents with mental health problems

**DOI:** 10.3389/fnint.2022.946842

**Published:** 2022-09-02

**Authors:** Mei Zhao, Mi Hu

**Affiliations:** ^1^School of Medicine, Hunan Polytechnic of Environment and Biology, Hengyang, China; ^2^Xiangya School of Public Health, Central South University, Changsha, China

**Keywords:** mental health problems, formal help-seeking, informal help-seeking, adolescents, influencing factors, China

## Abstract

**Objective:**

Mental health problems are highly prevalent among adolescents yet the utilization of mental health services among such a population is very low. This study was conducted to examine mental health problems and related help-seeking behaviors among a Chinese sample of adolescents.

**Methods:**

A total of 3,480 students were recruited from four middle- and high schools in Changsha City, Hunan province, and completed an online questionnaire that assessed their general information, mental health problems including depression, anxiety, self-harm, and suicide ideation, as well as their help-seeking behaviors from both formal (including psychological teachers and mental health professionals) and informal sources (including family, friends, and teachers).

**Results:**

The participants had a prevalence of 13.7% for depression, 11.5% for anxiety, 9.8% for self-harm, and 9.1% for suicide ideation. Although a high rate of help-seeking behaviors was observed (73.0%), most were concentrated in informal sources (99.3%), while only a small portion of participants resorted to formal sources (13.9%). Being female (OR: 1.45, 95% CI: 1.15–1.83), higher grade (OR: 1.32, 95% CI: 1.01–1.73), school mental health resources not available (OR: 1.39, 95% CI: 1.02–1.88), without suicide ideation (OR: 2.03, 95% CI: 1.42–2.90) were all associated with increased likelihood of formal help-seeking behaviors. On the other hand, complete middle school (OR: 0.36, 95% CI: 0.22–0.59), the middle level of academic ranking (OR: 0.64, 95% CI: 0.42–0.97), and higher father education levels (OR: 0.54–0.56, 95% CI: 0.33–0.90) were all associated with a decreased likelihood of formal help-seeking behaviors.

**Conclusion:**

Our results showed a higher prevalence of help-seeking behavior for emotional or psychological problems during the past year. Compared to the high rate of informal help-seeking behaviors, students showed a lower propensity to seek formal help for their mental health problems, which may be explained by individual-level, family-level, and school-level factors. Our findings provide important implications for the development and popularization of targeted, needs-based mental health promotion and education programs in the future.

## Introduction

Mental health problems mainly include mental disorders that meet the diagnostic criteria (The fourth edition of the Diagnostic and Statistical Manual of Mental Disorders, DSM-IV; The International Classification of Diseases, 10th Revision, ICD-10) and psychological symptoms that do not meet the diagnostic criteria but cause distress and social impairment.

Globally, adolescent mental health is an important public health problem. The prevalence of adolescent mental health problems is as high as 20.0% (WHO, 2012; Children Comissioners, 2016). Research shows that about 25.0% of adolescents suffer from depression and anxiety, the prevalence of lifelong self-harm is 12.1%, and the annual suicide ideation of adolescents is about 20% (Michaud and Fombonne, 2005; Murphy, 2013; Oksanen et al., 2017).

In China, mental health problems among adolescents are serious, the prevalence of adolescent mental health problems is about 36–42% (Qu et al., 2015; Han et al., 2017; Li et al., 2020; Luo et al., 2020; Wang et al., 2020). Research shows that 20% are at risk for depression, 6% are at risk for generalized anxiety (Jiang et al., 2022), the prevalence of self-harm distinctly ranges from 6.4 to 47.5%, and about 32.09% of children and adolescents reported suicidal ideation (Lang and Yao, 2018).

Mental health problems can negatively affect the psycho-social development of adolescents (WHO, 2018). The research shows that adolescents with mental health problems are at increased risk of high-risk behaviors, such as substance abuse, internet addiction, unprotective sexual behaviors, and violence, which eventually lead to a high risk of self-injury and even suicide (Miller et al., 2011; Winstanley et al., 2012; Myers et al., 2021). The long-term existence of mental health problems among adolescents will continuously affect their health and social functioning in their adult years (Kowalenko and Culjak, 2018).

An effective way to reduce the negative impact of mental health problems on teenagers is help-seeking. Help-seeking for mental health problems has been defined as “an adaptive coping process that is the attempt to obtain external assistance to deal with mental health concerns” (Rickwood and Thomas, 2012). Based on the sources of help, help-seeking for mental health problems can be generally classified into two types: formal sources, such as mental health professionals and psychological teachers, and informal sources, such as family and friends (Rickwood and Thomas, 2012). While the prevalence of poor mental health is increasing among young people, mental health help-seeking remains low. Low uptake of mental health services among adolescents has been a global health issue that is primarily attributed to their reluctance to seek help (Gulliver et al., 2010). Research shows that adolescents do not like to seek help from professional sources, which may be related to the fear of doctors and hospitals (Rickwood et al., 2007). At the same time, teenagers and their parents are also afraid of others knowing that they are suffering from mental health problems, so they are also unwilling to turn to friends or family (Gulliver et al., 2010). It has been well-acknowledged that adolescents most in need of mental health services are least likely to access them (Gulliver et al., 2010), that is what we need to discuss (Hunt and Eisenberg, 2010; D’Avanzo et al., 2012; Brown et al., 2014; Menberu et al., 2018).

Similarly, mental health service utilization in China is also very low. Low utilization rate was reported even in the most developed metropolitan cities, such as Beijing (2.7%) and Shanghai (3.1%) (Demyttenaere et al., 2004), as well as less developed areas, such as northwestern China (2.3%) (Liu et al., 2018). In addition, people with mental health problems were less likely to seek professional help; a study in four provinces showed that only 8% of participants had sought professional help (Phillips et al., 2009).

Various factors have been demonstrated to affect adolescents’ help-seeking behaviors for mental health problems, which may be generally classified into three levels: individual-level (such as age, gender, and grade), family-level (such as parental education, family income, and living environment), and school level (such as mental health-related policy, mental health promotion programs, and school-based mental health services) (Glascoe, 2003; Magaard et al., 2017; O’Reilly et al., 2018). Understanding influencing factors that affect adolescents’ help-seeking behaviors is fundamental for the recognition of various facilitators and barriers for help-seeking so as to inform targeted interventions and program development to solve their mental health problems. This study was conducted to examine mental health problems and help-seeking behaviors among a sample of middle school and high school students in Changsha City, Hunan Province of China. To be specific, we aimed to explore: (1) the prevalence of four common mental health problems: depression, anxiety, self-harm, and suicide ideation; (2) the prevalence of help-seeking behaviors for mental health problems from both formal and informal sources; and (3) the influencing factors of adolescents’ formal help-seeking behaviors.

## Materials and methods

### Participants and procedure

This was a cross-sectional study conducted among middle and high school students in Changsha City of Hunan Province from October 2016 to January 2017. The sample size of this study is calculated according to the research results of Brown J (the incidence of seeking help for mental health problems was 73.7%) under the confidence level of 95% and the error range of 5% (D’Avanzo et al., 2012; Brown et al., 2014; Menberu et al., 2018). And the minimum sample size for epidemiological investigation is 298. Our target population was students in Grades 7, 8, 10, and 11 (the junior high school consists of grades 7, 8, and 9, while the high school consists of grades 10, 11, and 12.). We did not include students in Grades 9 and 12 because they were under great academic pressure to prepare for the upcoming high school/college entrance examination and may not be able to participate in our survey. Changsha’s middle and high schools are divided into ordinary high schools, vocational high schools, ordinary middle schools, and complete middle schools (refers to a school with both junior high school and senior high school). A stratified multi-stage cluster sampling method was used to select middle- and high school students. In the first stage, four schools were selected from the urban area of Changsha, including one ordinary high school, one vocational high school, and two complete middle schools. In the second stage, all students in grades 7, 8, 10, and 11 were selected from these four middle and high schools. The final sample size of this study was 3,669. Eligible participants were required to be grades 7, 8, 10, and 11 students admitted to the above-mentioned four schools, and be able to read and write. Those who were unable to understand and communicate were excluded from our study.

The study was conducted online, with all questionnaires produced, distributed, and collected with the online survey tool Wenjuanxing during the computer classes of the students. The investigators were composed of teachers from those four schools, graduate students from the School of Public Health, Central South University, and undergraduate and graduate students from the educational psychology department of Hunan Normal University. Before the formal investigation, the investigators received at least two systematic and standardized training for the study to ensure internal consistency. During the study, there were four investigators (must include a psychology teacher and a computer teacher) in each computer classroom to assist the students to fill in the electronic questionnaire. Each questionnaire was submitted through the Wenjuanxing to check the quality and ensure no missing data. Among the 3,669 eligible students, 80 students refused to fill in the questionnaire, 29 were on sick leave during the survey day, and 80 could not submit the questionnaire due to network problems, leading to a final sample of 3,480 students who completed the questionnaires, with a response rate of 95%. Our team was responsible for producing the questionnaire, and the data was collected by the psychology teachers in these four schools. The research is based on secondary data without any personal information. Before the study, the psychology teacher informed the students about the content and purpose of the study and promised to keep their personal information strictly confidential. All respondents voluntarily agreed to participate in the study, provided written informed consent before participating in the study, and completed an online questionnaire by themselves.

### Measures

#### General information

General information, including individual-level, family-level, and school-level factors, was collected through self-report questionnaires. Individual-level factors included gender, grade, only-child, and academic ranking. Family-level factors included parental marital status, father education, mother education, family income, and availability of family mental health resources. School-level factors included school type and availability of school mental health resources.

#### Mental health problems

##### Depression

Depression was assessed by the Patient Health Questionnaire-9 (PHQ-9) (Spitzer et al., 1999), which includes nine items to screen for depressive symptoms in the past 2 weeks. Each item is rated on a 4-point Likert scale from 0–“not at all” to 3–“nearly every day.” The total score ranges from 0 to 27, with a higher score indicating more depressive symptoms and a cutoff of 10 distinguishing those with depression and those without. In this study, the PHQ-9 showed good internal consistency, with a Cronbach’s alpha of 0.83.

##### Anxiety

Anxiety was assessed by the Generalized Anxiety Disorder Scale-7 (GAD-7) (Spitzer et al., 2006), which included seven items to screen for anxiety symptoms in the past 2 weeks. Each item is rated on a 4-point Likert scale from 0–“not at all” to 3–“nearly every day.” The total score ranges from 0 to 21, with a higher score indicating more anxiety symptoms and a cutoff of 10 distinguishing those with anxiety and those without. In this study, the GAD-7 showed good internal consistency, with a Cronbach’s alpha of 0.86.

##### Self-harm

Self-harm was assessed by one self-designed item “Have you intentionally hurt yourself in the past year,” with those answering “yes” identified as positive for self-harm.

##### Suicide ideation

Suicide ideation was assessed by one self-designed item “In the past year, have you seriously considered suicide,” with those answering “yes” identified as positive for suicide ideation.

#### Help-seeking behaviors for mental health problems

Help-seeking behaviors for mental health problems were assessed by five self-report questions asking whether the respondents had sought any help due to mental health problems during the past year from five different sources: family, friends, teachers, psychological teachers, and mental health professionals. We further classified the five sources into two major types: formal sources and informal sources. Participants who had sought help from any of the following three sources: family, friends, and teachers for mental health problems were identified as having sought informal help; while participants who had sought help from either psychological teachers or mental health professionals were identified as having sought formal help.

### Statistical analysis

Descriptive statistics were used to summarize all study variables. Continuous variables were described using mean and standard deviation, and categorical variables were described using frequency and percentage. χ^2^ test was used to compare the individual-level, family-level, and school-level differences between those seeking help and those who did not seek help for mental health problems, followed by a multivariate logistic regression analysis to identify influencing factors of formal help-seeking for mental health problems. Since the dependent variable is whether there is a behavior of seeking help, which is a binary variable, and the related independent variables include multiple measurement data and enumeration data, our study constructs a logistic regression model to predict it. All data were analyzed using SPSS version 23.0. Values of *p* < 0.05 were considered statistically significant (two-tailed test).

## Results

### General information

Table 1 shows the general information of the participants that included individual-level, family-level, and school-level characteristics. For individual-level factors, slightly over half of the participants were male (50.9%) and only-child (54.8%), most were in high grades of 10 and 11 (69.0%), middle level for academic ranking (68.5%). For family-level factors, most families were in stable marital status (90.2%) with family mental health resources available (82.2%), and the largest proportion of parental education was high school and below for both fathers (55.9%) and mothers (62.5%), family income levels were concentrated in the 2,000–5,000 Yuan/month group (30.8%) and 5,000–1,000 Yuan/month group (36.8%). For school-level factors, 61.7% of the participants were in complete middle school, and 75.8% of them were in school with mental health services available.

**TABLE 1:** General information of the participants (*n* = 3,480).

Variables	Category	*n*	%
Individual-level factors			
Gender	Male	1,773	50.9
	Female	1,707	49.1
Grade	Lower (7 and 8)	1,080	31.0
	Higher (10 and 11)	2,400	69.0
Only-child	Yes	1,907	54.8
	No	1,573	45.2
Academic ranking	Top 10	705	20.3
	Middle level	2,386	68.5
	Last 10	389	11.2
Family-level factors			
Parental marital status	Married	3,140	90.2
	Divorced	304	8.7
	Widowed	36	1.1
Father education	High school and below	1,945	55.9
	College	1,289	37.0
	Master and above	246	7.1
Mother education	High school and below	2,174	62.5
	College	1,155	33.2
	Master and above	151	4.3
Family income (Yuan/month)	<2,000	153	4.4
	2,000–5,000	1,072	30.8
	5,001–10,000	1,282	36.8
	>10,000	973	28.0
Available family mental health resources	Yes	2,860	82.2
	No	620	17.8
School-level characteristics			
School type	Ordinary high schools	1,062	30.5
	Complete middle schools	2,147	61.7
	Vocational high schools	271	7.8
Available school mental health resources	Yes	2,637	75.8
	No	843	24.2

### Mental health problems

Table 2 shows the prevalence of four major types of mental health problems among the participants. Among the 3,480 participants, 477 were screened positive for depression (13.7%), 401 were screened positive for anxiety (11.5%), 341 reported self-harm behaviors in the past year (9.8%), and 316 reported suicide ideations in the past year (9.1%).

**TABLE 2:** Mental health problems (*n* = 3,480).

Variables	Categories	*n*	%
Depression	Yes	477	13.7
	No	3,003	86.3
Anxiety	Yes	401	11.5
	No	3,079	88.5
Self-harm	Yes	341	9.8
	No	3,139	90.2
Suicide ideation	Yes	316	9.1
	No	3,164	90.9

### Help-seeking behaviors

Table 3 shows the pattern of participants’ help-seeking behaviors. Among the 3,480 participants, 2,539 (73.0%) had help-seeking behavior for emotional or psychological problems during the past year. The number of participants with depression, anxiety, self-harm, and suicidal ideation were 477, 401, 341, and 274, respectively, of which 367 (76.9%), 317 (79.1%), 274 (80.4%), and 247 (77.2%) had help-seeking behavior. After stratifying sources of help by formal and informal sources, among the 2,539 participants with help-seeking behavior, 99.3% turned to informal sources [friends (85.8%), family (64.5%), and teachers (24.1%)], 13.9% sought formal sources [psychological teachers (10.2%) and mental health professionals (8.3%)]. Detailed information on the sources of help-seeking for people with the four psychological problems is available in Table 3, these people tend to seek help from friends in informal sources.

**TABLE 3:** Help-seeking behaviors and help-seeking sources.

Variables	Categories				
Help-seeking behaviors	In total (3,480)	Depression (477)	Anxiety (401)	Self-harm (341)	Suicide ideation (316)
Yes	2,539 (73.0%)	367 (76.9%)	317 (79.1%)	274 (80.4%)	244 (77.2%)
No	941 (27.0%)	110 (23.1%)	84 (20.9%)	67 (19.6%)	72 (22.8%)
**Patterns of help-seeking behaviors among those who sought help**					
	In total (2,539)	Depression (367)	Anxiety (317)	Self-harm (274)	Suicide ideation (244)
Formal sources	352 (13.9%)	66 (18.0%)	54 (17.0%)	47 (17.1%)	54 (22.1%)
Psychological teachers	260 (10.2%)	52 (14.2%)	39 (12.3%)	31 (11.3%)	38 (15.6%)
Mental health professionals	211 (8.3%)	40 (10.9%)	35 (11.0%)	32 (11.7%)	37 (15.2%)
Informal sources	2,521 (99.3%)	361 (98.4%)	313 (98.7%)	272 (99.2%)	239 (97.0%)
Friends	2,181 (85.8%)	305 (83.1%)	248 (78.2%)	224 (81.8%)	200 (82.0%)
Family	1,640 (64.5%)	217 (59.1%)	191 (60.3%)	160 (58.4%)	134 (54.9%)
Teachers	613 (24.1%)	91 (24.8%)	82 (25.9%)	72 (26.3%)	56 (23.0%)

### Number of help-seeking sources

Table 4 shows the number of help-seeking sources. As for the number of persons they sought help from, among the 2,539 participants with help-seeking behaviors, 39.7% only sought help from 1 type (1.2% sought help from formal sources), and 37.3% sought help from 2 types (5.9% sought help from formal sources), 23.0% of the people sought help from 3 or more types (48.6% sought help from official sources). See Table 4 for details on how many types of sources people with the four psychological problems seek help from, and these people tend to seek help from one type of source from informal sources.

**TABLE 4:** Number of help-seeking object.

Variables	*N*	Formal sources (%)	Informal sources (%)
**In total (*n* = 2,539)**			
1	1,007 (39.7%)	12 (1.2)	995 (98.8)
2	948 (37.3%)	56 (5.9)	942 (99.4)
≥ 3	584 (23.0%)	284 (48.6)	584 (100.0)
**Depression (*n* = 367)**			
1	161 (43.9%)	3 (1.9)	158 (98.1)
2	120 (32.7%)	17 (14.2)	117 (97.5)
≥ 3	86 (23.4%)	46 (53.5)	86 (100.0)
**Anxiety (*n* = 317)**			
1	124 (39.1%)	1 (0.8)	123 (99.2)
2	116 (36.3%)	14 (12.1)	113 (97.4)
≥ 3	77 (24.3%)	39 (50.6)	77 (100.0)
**Self-harm (*n* = 274)**			
1	114 (41.6%)	0 (0.0)	114 (100.0)
2	92 (33.6%)	9 (9.8)	90 (97.8)
≥ 3	68 (24.8%)	38 (55.9)	68 (100.0)
**Suicide ideation (*n* = 244)**			
1	95 (38.9%)	2 (2.1)	93 (97.9)
2	86 (35.3%)	10 (11.6)	83 (96.5)
≥ 3	63 (25.8%)	42 (66.7)	63 (100.0)

The help-seeking sources include formal sources (psychological teachers and mental health professionals), and informal sources (friends, family, and teachers).

### Influencing factors of formal help-seeking behaviors

Considering the relatively low prevalence of formal help-seeking behaviors, we further run a multivariate logistic regression to explore the influencing factors of formal help-seeking behaviors by including all general information and participants’ mental health problems as independent variables. The results are shown in Table 5; among the 15 included factors, 7 factors remained significant after controlling for all the other factors: gender, grade, academic ranking, father education, school type, availability of school mental health resources, and suicide ideation. Being female (OR: 1.45, 95% CI: 1.15–1.83), higher grade (OR: 1.32, 95% CI: 1.01–1.73), school mental health resources not available (OR: 1.39, 95% CI: 1.02–1.88), without suicide ideation (OR: 2.03, 95% CI: 1.42–2.90) were all associated with increased likelihood of formal help-seeking behaviors. On the other hand, complete middle school (OR: 0.36, 95% CI: 0.22–0.59), middle level of academic ranking (OR: 0.64, 95% CI: 0.42–0.97), higher father education levels (OR: 0.54–0.56, 95% CI: 0.33–0.90) were all associated with a decreased likelihood of formal help-seeking behaviors.

**TABLE 5:** Multivariate logistic regression of influencing factors of formal help-seeking behaviors.

Variables	Category	OR (95%CI)	*p*
**Individual-level factors**			
Gender	Male		Ref
	Female	**1.45 (1.15, 1.83)**	**0.002**
Grade	Lower (7 and 8)		Ref
	Higher (10 and 11)	**1.32 (1.01, 1.73)**	**0.048**
Only-child	Yes		Ref
	No	0.88 (0.69, 1.12)	0.283
Academic ranking	Top 10		Ref
	Middle level	**0.64 (0.42, 0.97)**	**0.034**
	Last 10	0.77 (0.54, 1.08)	0.129
**Family-level factors**			
Parental marital status	Married	Ref	
	Divorced	1.04 (0.30, 3.62)	0.95
	Widowed	1.31 (0.35, 4.96)	0.69
Father education	High school and below	Ref	
	College	**0.54 (0.33, 0.90)**	**0.016**
	Master and above	**0.56 (0.35, 0.90)**	**0.015**
Mother education	High school and below	Ref	
	College	0.69 (0.37, 1.28)	0.314
	Master and above	0.92 (0.51,1.65)	0.298
Family income (Yuan/month)	<2,000	Ref	
	2,000–5,000	1.33 (0.76,2.34)	0.314
	5,001–10,000	1.24 (0.91, 1.71)	0.178
	>10,000	1.09 (0.81, 1.47)	0.585
Available family mental health resources	Yes	Ref	
	No	1.23 (0.87, 1.74)	0.241
**School-level factors**			
School type	Ordinary high schools	Ref	
	Complete middle schools	**0.36 (0.22, 0.59)**	**<0.001**
	Vocational high schools	0.89 (0.57, 1.39)	0.611
Available school mental health resources	Yes	Ref	
	No	**1.39 (1.02, 1.88)**	**0.038**
**Mental Health Problems**			
Depression	Yes	Ref	
	No	0.74 (0.51, 1.06)	0.102
Anxiety	Yes	Ref	
	No	0.85 (0.57, 1.25)	0.405
Self-harm	Yes	Ref	
	No	0.73 (0.47, 1.14)	0.271
Suicide ideation	Yes	Ref	
	No	**2.03 (1.42, 2.90)**	**<0.001**

Bold indicates that the difference is statistically significant.

## Discussion

### Summary of findings

The main findings of the study are that the participants had a prevalence of 13.7% for depression, 11.5% for anxiety, 21.0% for self-harm, and 9.1% for suicide ideation. Nearly three-quarters of the participants had sought help for their emotional or psychological problems during the past year (73.0%). Among the participants who had help-seeking behaviors, most turned to informal sources (99.3%), such as family, friends, and teachers for help, while only a small portion of participants resorted to formal sources (13.9%), such as psychological teachers and mental health professionals. Being female, having higher grades, school mental health resources not available, without suicide ideation were all associated with an increased likelihood of formal help-seeking behaviors. On the other hand, complete middle school, the middle level of academic ranking, and higher father education levels were all associated with a decreased likelihood of formal help-seeking behaviors.

### Mental health problems

In this study, the prevalence of depression and anxiety is similar to the results of a recent study conducted on a large sample of adolescents from China (Mao et al., 2019; Cao et al., 2022), and the prevalence of self-harm is similar to the results of a recent study conducted among middle school students in Shanghai and middle school students in rural areas of Anhui province (Chen et al., 2013; Zhang et al., 2019), and the prevalence of suicidal ideation is much lower than the results of a recent study conducted in a large sample of children and adolescents in China (Tan et al., 2018). The differences in the prevalence of various mental problems in various studies may be explained by multiple factors, such as study location, samples, measurement of mental health problems including the scales used and the time periods assessed, which all contributed to the large diversity of the study results.

### Help-seeking behaviors

Although our study showed a high prevalence of help-seeking behaviors among middle and high school students, most of their help-seeking sources have been concentrated in informal sources, while much fewer participants turned to formal sources for help. This finding was generally consistent with the past literature showing higher utilization rates of informal resources ranged from 60 to 70%, than that of formal resources that ranged from 25 to 40% (D’Avanzo et al., 2012; Brown et al., 2014; Menberu et al., 2018). The high propensity of participants to seek help from informal sources may reflect the public’s attitude toward treating mental health problems such as depression and anxiety as some normal emotional reactions to adverse life events instead of a mental illness that needs professional treatment (Holzinger et al., 2011). Interpretation of “feeling mentally poor” and “psychological difficulty” as not having an illness may lead to a lower propensity to seeking professional help from psychological teachers or mental health professionals. Another possible explanation may be the high stigma attached to mental health problems may prohibit participants from seeking professional help from outside due to fear of privacy disclosure and being discriminated (Schnyder et al., 2017). As a result, participants may prefer to turn to close acquaintances who know them better and are able to protect their privacy. Also, the low rate of help-seeking from formal sources may indicate the low accessibility or awareness of the participants to such resources, which may inform the development and advertisement of mental health promotion intervention programs in schools.

### Influencing factors of formal help-seeking

Three individual-level factors have been identified as influencing participants’ formal help-seeking behaviors: gender, grade, and academic ranking. The finding that females were more likely to seek formal help than males is consistent with the bulk of evidence showing higher barriers in males for help-seeking, which may be explained by their poor mental health literacy and greater alignment with norms of hegemonic masculinity (Yu et al., 2015; Clark et al., 2020). Students in the higher grades were more likely to seek formal help than students in the lower grades, which may be explained by the relatively higher education level and as a result higher mental health literacy in higher grade students (Magaard et al., 2017). Similarly, students in the middle level of the academic ranking were less likely to seek formal help than students in the top 10, which may also be explained by their relatively lower level of mental health literacy (Magaard et al., 2017). One family-level factor has been identified as influencing participants’ formal help-seeking behaviors: father education. The finding that students with a higher level of father education were less likely to seek formal help may be explained by the fact that they could get enough support from their family and thus need not turn to professional help, which needs to be explored further. Two school-level factors have been identified as influencing participants’ formal help-seeking behaviors: school type and available school mental health resources. Students with unavailable school mental health resources were more likely to seek formal help and students from complete middle school were less likely to seek formal help. These results indicate that mental health promotion and education programs are more needed in complete middle schools and schools without mental health resources. Finally, among the four types of mental health problems, only suicide ideation was associated with formal help-seeking. Participants without suicide ideation were more likely to seek formal help than those with suicide ideation, indicating the need for active screening students at high risk of suicide ideation and providing timely and professional mental health services to them at school.

Several limitations need to be taken into consideration before interpreting the study results. First, the assessment of mental health problems was based on some simple screening scales or questions and limited to only four types, which may limit the accuracy and comprehensiveness of mental health assessment. Future standard assessment tools that cover a wide range of mental health diagnoses may be needed for a better and more comprehensive measurement of mental health problems. Second, the assessment of help-seeking behaviors in the past year relied on participants’ self-reports, which may be subject to recall bias. Future studies should consider adding more objective indicators such as medical records for formal help-seeking to get a more accurate assessment of their actual help-seeking behaviors. Third, the cross-sectional study design may preclude any causal relationship to be established between influencing factors and formal help-seeking. Future longitudinal study design may be needed to confirm causal relationships.

## Conclusion

Our results showed that the prevalence of mental health problems was generally lower in this study than that reported in most previous similar studies in other areas of China, which may be explained by multiple research factors. Although students showed a high prevalence of help-seeking behaviors for mental health problems, most students turned to informal sources instead of formal sources. Compared to the high rate of informal help-seeking behaviors, students showed a lower propensity to seek formal help for their mental health problems, which may be explained by individual-level, family-level, and school-level factors. Our findings provide important implications for the development and popularization of targeted, needs-based mental health promotion and education programs in the future.

## Data availability statement

The original contributions presented in this study are included in the article/supplementary material, further inquiries can be directed to the corresponding author.

## Author contributions

MH: conceptualization and methodology. MZ: investigation and data analysis. MH and MZ: writing. Both authors contributed to the article and approved the submitted version.
